# Skilled for Whom? Immigration Policy, Racial Capitalism, and the Reproduction of Inequality in Britain

**DOI:** 10.1111/1468-4446.70101

**Published:** 2026-03-02

**Authors:** Muhammad Abdul Aziz, Muhammad Umar, Deborah Hamer‐Acquaah

**Affiliations:** ^1^ University of Greenwich London UK; ^2^ University of Hertfordshire Hatfield UK; ^3^ Bradford Academy Bradford UK

**Keywords:** higher education, immigration policy, labour market, racial capitalism

## Abstract

This paper examines the UK's 2025 *Immigration White Paper* as a critical site for understanding how immigration policy functions as an instrument of racial capitalism. Drawing on Critical Race Theory, the theory of social reproduction, and intersectionality, it interrogates how the state's construction of the ‘skilled migrant’ operates as a racially coded category that privileges whiteness, anglocentric credentials, and neoliberal norms of value. Rather than treating the White Paper as a discrete policy episode, the analysis situates it within a longer genealogy of immigration governance that reproduces structural inequalities across higher education and graduate employment. By tracing how migrant ‘worthiness’ is encoded through racialised and classed proxies—such as language fluency, academic credentials, and salary thresholds—the paper contributes to wider sociological debates on bordering, credentialism, and state racial formation. It demonstrates that the British state's discourse of ‘merit’ and ‘skill’ is inseparable from exclusionary practices that undermine the promise of equal opportunity for racialised citizens and migrants alike. The paper concludes by advancing a forward‐looking framework for understanding policy as both a site of intervention and a generator of symbolic and material hierarchies.

## 1 Introduction

In May 2025, the UK government released a White Paper titled *Restoring Control over the Immigration System*, outlining its intent to significantly reduce net migration and tighten access across work, study, and family pathways (HM Government 2025). Although framed as a technocratic recalibration of border policy, this intervention is embedded within broader political currents that elevate national sovereignty, cultural conformity, and economic productivity as benchmarks of migrant worth. These developments unfold within a domestic context in which racial and ethnic inequalities remain structurally entrenched in both higher education and the labour market (Heath and Di Stasio 2019; Zwysen et al. 2021; Foster and George 2025). Consequently, the White Paper's invocation of ‘merit,’ ‘contribution,’ and ‘integration,’ articulated through bureaucratic languages of fairness and control, operates as a discursive mechanism through which racialised hierarchies are legitimised and reproduced.This paper situates the 2025 White Paper within a lineage of British immigration policies that have operated as de facto instruments of racial ordering (Goodfellow 2020; Gentleman 2024). Drawing on CRT, intersectionality, and social reproduction theory, the analysis interrogates how immigration controls—ostensibly targeting legal status or skills—function as symbolic and material mechanisms of racial capitalism.CRT foregrounds the permanence of institutional racism and the adaptability of white supremacy within liberal democratic states (Delgado and Stefancic 2023); intersectionality reveals how race, class, and gender co‐produce differentiated vulnerabilities (Crenshaw 1991); and Bourdieu offers tools to understand how institutionalised capital—credentialed, cultural, and linguistic—is valorised unequally, thereby reproducing stratification under the guise of fairness (Bourdieu 1986).

The political terrain on which these policies operate remains sharply divided. In the aftermath of the Black Lives Matter protests, the UK Government's Commission on Race and Ethnic Disparities (CRED) report, widely known as the Sewell Report, denied the existence of institutional racism and instead attributed disparities to cultural and familial variables (Commission on Race and Ethnic Disparities (CRED) 2021). This deflection of structural critique has been fiercely contested. Scholars and practitioners point to compelling empirical evidence—including differential graduate outcomes (Zwysen et al. 2021), hiring discrimination (Heath and Di Stasio 2019), and educational disparities (Seuwou et al. 2023)—to argue that racial injustice remains systemic. The United Nations' condemnation of the CRED report for ‘normalising white supremacy’ (Mohdin 2021) further underscores the global resonance of Britain's racial discourse.

Amidst this ideological fracture, the 2025 immigration reforms risk deepening the exclusions they purport to regulate. A suppressed Home Office review, published belatedly in 2024, confirmed that past immigration laws were explicitly designed to limit settlement by individuals ‘with black or brown skin’ (Gentleman 2024), thereby reinforcing racial hierarchy through policy. Although contemporary rhetoric emphasises skills and self‐sufficiency, these criteria are encoded through classed and racialised proxies: salary thresholds, English fluency, and credential equivalence. As seen in the ‘hostile environment’ policies and the Windrush scandal, such metrics disproportionately harm racialised Britons and migrants alike (Eddo‐Lodge 2019; Williams 2020). This paper contends that the latest reforms, while articulated through bureaucratic and managerial language framing restriction as fairness and control as responsibility, reproduce these patterns by rearticulating immigration policy as a site of racialised state formation.

### 1.1 Theoretical Framework

This paper draws on three intersecting theoretical lenses—Critical Race Theory (CRT), intersectionality, and theory of social reproduction—to interrogate how immigration policy, particularly the 2025 White Paper, functions as a mechanism of racialised differentiation and inequality. This analysis is guided by the broader conceptual framing of racial capitalism, operationalised through these three lenses, to examine how migration governance reproduces symbolic and material hierarchies. These frameworks illuminate how the bureaucratic and moral vocabularies of policy—invoking fairness, skill, and contribution—function as instruments through which exclusion and hierarchy are normalised and reproduced.

#### 1.1.1 Critical Race Theory (CRT)

Offers a foundational critique of the liberal assumptions underpinning British publick discourse. It posits that racism is not an aberration but a permanent and structuring feature of social institutions, including those presumed to be race‐blind (Gillborn 2008). CRT insists that systemic racism persists despite formal equalities, manifesting in persistent outcome disparities between white and minoritised groups across education and employment (Heath and Di Stasio 2019; Pilkington 2020; Arday et al. 2022). In UK universities, for example, institutional whiteness renders Black Asian and Minority Ethnic BAME students hyper‐visible as racialised subjects while simultaneously marginalising their experiences—producing awarding gaps and undermining their sense of belonging (Gillborn 2008; Bhopal 2024b). In the labour market, hiring discrimination remains a consistent finding across field experiments (Heath and Di Stasio 2019), while ‘colour‐blind’ policies frequently obscure the structural conditions under which minoritised graduates attempt to succeed. CRT therefore interrogates how whiteness, as a normative framework, shapes who counts as deserving, meritorious, or employable (Gillborn 2008; Tikly 2022).

#### 1.1.2 Intersectionality

Originating in Black feminist thought (Crenshaw 1991), deepens this analysis by rejecting additive or one‐dimensional approaches to inequality. It foregrounds the mutually constitutive dynamics of race, gender, and class, revealing how ostensibly technocratic or fairness‐based policies can differentially impact individuals across intersecting lines of marginalisation (Carastathis 2014). For example, recent research documents how ethnic minority women face compounded barriers in graduate recruitment, with some groups experiencing both gendered and racialised penalties in hiring and career progression (Zwysen and Longhi 2018; Dickson et al. 2024). Class position further shapes these outcomes, as access to elite networks, credentialled capital, and ‘soft skills’ often reflect middle‐class norms (Ingram and Allen 2019). Intersectionality also challenges the essentialising tendencies of terms like ‘BAME’ by disaggregating the diverse—and at times divergent—experiences of different minority groups. In this light, the Sewell Report's binary framing of either race or class as causal mechanisms is not only analytically flawed but politically expedient in its denial of structural complicity (Hall et al. 1978; Tikly 2022).

#### 1.1.3 Theory of Social Reproduction

Complements these perspectives by offering a sociological account of how inequality is perpetuated through symbolic, cultural, and institutional practices (Bourdieu 1986; Bourdieu and Passeron 1990). In education and employment, institutions valorise specific forms of cultural capital—linguistic fluency, institutional prestige, and confident self‐presentation—that often correlate with whiteness and middle‐class habitus (Ingram and Allen 2019; Burke et al. 2020). These markers are misrecognised as indicators of intrinsic merit, thereby reproducing exclusion through bureaucratic and cultural practices that appear objective but operate along racialised and classed lines. Migrants and minoritised graduates lacking such capital are routinely deemed less ‘employable,’ regardless of formal qualifications (Zwysen and Longhi 2018; Oputa and Cross 2021). Social capital, including informal networks and mentorship access, is likewise stratified, constraining mobility even further. Bourdieu's concept of habitus elucidates how repeated exposure to exclusion may lead individuals to internalise a sense of unbelonging, thusly reinforcing inequality at both psychological and institutional levels (Bourdieu and Passeron 1990; Backer and Cairns 2021).

Taken together, these frameworks enable immigration policy to be conceptualised not merely as a technocratic border tool but as a discursive and institutional apparatus through which the state regulates belonging, merit, and racialised value. In the case of the 2025 White Paper, salary thresholds, language requirements, and family reunification criteria do not simply filter by skill or need—they also encode long‐standing racial hierarchies through bureaucratic languages of fairness and responsibility that legitimise exclusion.

## 2 Methodology

This paper employs a Critical Discourse Analytic (CDA) approach to interrogate the *Immigration White Paper: Restoring Control over the Immigration System* (HM Government 2025) as a key policy artefact in the racialised reproduction of ‘skills’ discourse. CDA is particularly suited to the sociological study of state policy because it foregrounds how institutional texts reproduce ideology and power through bureaucratic and managerial languages of fairness, efficiency, and control (Fairclough 2013; Wodak 2001; van Dijk 1993).It enables the linking of textual form to social practise by tracing how linguistic and rhetorical choices construct legitimacy, belonging, and exclusion. The *White Paper* was selected as the primary dataset because of its centrality to Britain's post‐Brexit migration regime and its explicit claim to restore ‘control’ through a re‐ordering of skill and value.

The analysis proceeded through a close, theory‐informed reading of the document's ministerial forewords, statistical framings, and policy chapters, supported by relevant parliamentary debates and ministerial statements referenced within the text. Attention was given to the *lexical*, *metaphorical*, and *categorical* structures through which migrants are constructed as productive or problematic. Coding was iterative, moving between deductive theoretical categories derived from CRT (Crenshaw 1991; Delgado and Stefancic 2023), intersectionality, and Bourdieu's notions of *capital*, *field* and *symbolic violence*, and inductive themes that emerged from recurring textual patterns and silences. Key thematic clusters included *merit and worthiness*, *mobility and containment*, and *institutional coupling* between higher education, labour market policy, and border control.

Rather than seeking empirical generalisation, this interpretive approach reveals how discourse functions as a racialising technology—translating social hierarchies into apparently objective classifications of skill and value. The analysis therefore situates the 2025 policy within longer genealogies of racial capitalism and post‐imperial governance (Bhattacharyya 2018; Shilliam 2018), illuminating the mechanisms through which the state's managerial rationality sustains racialised inequality under the banner of merit, productivity, and control.

While the White Paper represents a recent and still‐developing policy framework, the analysis is necessarily prospective. The purpose here is not to evaluate its empirical outcomes, which will unfold over time, but to identify the policy's logics and likely consequences as they are inscribed in its textual architecture. These interpretations are grounded in historical precedent and enduring structural inequalities within Britain's immigration and higher education systems, ensuring that the analysis remains interpretively rigorous rather than speculative.

### 2.1 Analysis: The 2025 Immigration White Paper as a Racialised Policy Apparatus

The 2025 White Paper, *Restoring Control over the Immigration System* (HM Government 2025), articulates a strategic recalibration of UK immigration policy, framed through the language of sustainability, fairness, and integration. While presented as a technocratic intervention, its underlying logick reflects a broader political commitment to economic utilitarianism and nationalist identity consolidation. The assertion that net migration ‘must come down’ (HM Government 2025: Ch.1) is not simply a demographic concern—it is a symbolic act of boundary maintenance, rooted in long‐standing anxieties about race, labour, and belonging. The paper reaffirms the idea that migrant presence is justifiable only insofar as it yields tangible economic value and cultural conformity—an ethos that reinforces exclusionary hierarchies under the guise of merit and contribution.

A central target of the reform agenda is the reconfiguration of work‐related migration. The proposed elevation of the skill threshold for visas to Regulated Qualifications Framework (RQF) Level 6 (equivalent to a UK Bachelor's degree) and increased salary requirements (HM Government 2025) reasserts a narrow valuation of human capital. These measures stratify migrants not only by educational attainment, but also by proximity to dominant norms of professional legitimacy—standards shaped by classed and racialised proxies such as British‐accredited degrees and command of anglocentric communication styles. The creation of a Temporary Shortage Occupation List—applicable to lower‐skilled jobs with severe labour shortages—further institutionalises a bifurcated migration regime. Migrants filling these roles will be rendered disposable: admitted on time‐limited visas, denied family reunification rights, and excluded from settlement pathways. This logick echoes the cyclical importation of labour historically reserved for colonial and postcolonial subjects, reinforcing a racialised division of labour while cloaked in the bureaucratic language of ‘workforce planning’ and economic efficiency (Bhopal 2018; Bhambra et al. 2018).The rhetorical strategy of the White Paper oscillates between pragmatism and paternalism. On one hand, it claims to protect migrant workers from exploitation and raise wages for domestic workers; on the other, it frames integration as a one‐way obligation. Proposals to link settlement rights to employment continuity, tax contributions, and heightened English language requirements (HM Government 2025) reinscribe the conditionality of migrant belonging. These provisions position citizenship as an earned status reserved for those who demonstrate not only economic utility but also cultural assimilation. As Bourdieu (1986) and Bourdieu and Passeron (1990) would argue, the state here functions as a gatekeeper of symbolic capital—rewarding those whose habitus aligns with normative models of success, while marginalising others through bureaucratic ideals of fairness and merit that masque structural exclusion.

The White Paper's proposals concerning student and graduate migration further illuminate how economic valuation intersects with racialised governance. While international students are acknowledged for their revenue‐generating role—contributing over £12 billion in 2022/23 (Lewis and Bolton 2024), and an estimated £28.8 billion to the wider UK economy (Universities UK 2024)—the text quickly pivots to suspicion.

A surge in student dependants, particularly amongst cohorts from Nigeria, India, and Pakistan, was framed in the White Paper as a challenge to integration and social cohesion—an interpretation that racialises certain nationalities under the guise of technocratic concern (HM Government 2025). This discursive move implicitly links linguistic difference to social unfitness, feeding into broader tropes of cultural incompatibility. The tightening of Graduate Route provisions—hinting at shortened visa durations or economic contribution benchmarks—again signals the conditional welcome extended to racialised others. What is marketed as ‘responsible recruitment’ is, in practise, a demand that universities act as instruments of immigration enforcement, filtering students not only by academic merit but also by their projected economic assimilation — that is, their anticipated contribution to the national economy as measured through employability or income potential (Universities UK 2024).

These policies, while not explicitly racialised, operate as racialising technologies. They encode worthiness through performance metrics that disproportionately advantage white, middle‐class, Western‐coded migrants—such as those from Anglophone and Northern European countries—whose linguistic fluency, educational capital, and cultural proximity to British norms are read as evidence of integration and employability (HM Government 2025; Bhattacharyya 2018; Erel 2019). From a CRT perspective (Gillborn 2008; Delgado and Stefancic 2023), these reforms deploy bureaucratic and technocratic languages of fairness and merit through which structural inequalities are reproduced via proxies like salary bands and credential hierarchies. Intersectionality further sharpens the analysis: measures such as English requirements for dependants and the restriction of family reunification disproportionately affect lower‐income, female, and Global South migrants (Crenshaw 1991; Dickson et al. 2024). These individuals are not just subjected to economic precarity but are also rendered morally suspect, unassimilable, and disposable — a process documented in policy and institutional responses to racialised migration (Bhopal 2018; Trades Union Congress (TUC) 2023).

Thusly, the 2025 White Paper is not merely a document of migration management; it is a site of racial statecraft. Through the naturalisation of economic productivity and integration as criteria for inclusion, the British state enacts a form of racial capitalism that valorises select migrants while systematically marginalising others. This bifurcation of migrant desirability—along lines of class, race, and nation—raises urgent sociological questions about how policy operates as a covert mechanism of exclusion. As with the ‘hostile environment’ and Windrush scandal before it, the legacies of racial governance remain sedimented in the architecture of contemporary immigration reform (Williams 2020; Gentleman 2024).

### 2.2 Higher Education as a Racialised Site: Implications of the 2025 Immigration White Paper

#### 2.2.1 International Students, Bordering Logics, and the Racialised University

The 2025 Immigration White Paper's proposals to restrict student migration—particularly through limiting dependants and tightening post‐study work rights—carry significant implications for the racial and institutional composition of UK higher education. UK universities operate at the nexus of global mobility and national immigration controls, educating domestic students, including many from racially minoritised backgrounds, while also hosting large cohorts of international students and staff. A substantial proportion of these international students originate from Asian and African countries—such as India, China, Nigeria, Pakistan, and Bangladesh—bringing with them not only economic resources but also crucial racial and cultural diversity to British campuses (HESA 2025).

However, diversity is not simply additive; its effects are shaped by the institutional structures into which it enters. CRT scholars argue that universities are not objective or impartial terrains but are characterised by ‘institutional whiteness’ that regulates inclusion through dominant norms of cultural capital, linguistic legitimacy, and social networks (Pilkington 2020; Arday et al. 2022; Bhopal 2024b). Within this context, international students' presence can serve as a challenge to Eurocentric epistemologies and social norms—broadening classroom dialogue, unsettling stereotypes, and fostering intercultural exchange (Leask 2009; Aw et al. 2023). Yet immigration reforms that render the UK a less viable or hospitable destination—for instance, by barring spouses and children or by restricting post‐study work options—are likely to deter precisely those students whose visibility contests the normative whiteness of campus life.

Historical precedent reinforces this point. The 2012 abolition of the post‐study work visa precipitated a sharp decline in Indian student enrolments, which fell from 39,000 in 2010–11 to around 16,500 by 2016, as students turned to countries offering clearer pathways to employment and settlement. Current proposals may produce a similar effect, especially amongst students from the Global South, thereby reducing campus diversity and reinforcing the racial homogeneity of academic spaces. As studies have shown, even current levels of diversity have not translated into epistemic inclusion: Taylor et al. (2021) found that only 7% of authors on sampled life sciences reading lists were from BAME backgrounds, while Wong et al. (2021) argue that efforts at decolonisation remain partial and contested. Fewer international students may lessen institutional pressure to diversify curricula, staff demographics, and campus culture, particularly if the vocal student constituencies driving equity agendas are diminished.

This dynamic also has material implications. International tuition fees cross‐subsidise a wide range of university programmes, including equity initiatives that disproportionately benefit domestic minority ethnic students—such as mentorship schemes, targeted career support, and mental health services (Arday et al. 2022). Should international enrolments decline due to tighter visa policies, universities may find themselves with fewer resources to invest in these services. The 2025 White Paper's reductive approach to net migration—treating students as part of the ‘problem’ to be controlled—therefore risks undermining the very institutions attempting to redress racial disparities in education (HM Government 2025).

From a Bourdieusian perspective, these policies can be seen as reinforcing symbolic violence. Universities valorise particular forms of cultural and linguistic capital—often racialised and classed—which marginalised students may not possess or may be penalised for displaying differently (Bourdieu 1986; Ingram and Allen 2019). Immigration criteria that reward English fluency, economic self‐sufficiency, and assimilation into dominant norms map onto these institutional preferences, aligning border control with the reproduction of academic inequality. In this sense, immigration and education policy are co‐constitutive: they produce a shared racialised order of value, determining who belongs, who succeeds, and on what terms.

#### 2.2.2 Awarding Gaps and the Limits of Institutional Reform

The intersection between immigration discourse and educational inequality is further illuminated through the longstanding racial disparities in academic attainment—what is often referred to as the BAME awarding gap. Black Caribbean, Black African, Pakistani, and Bangladeshi students remain significantly less likely to achieve first‐ or upper‐second‐class degrees than their white counterparts, even when entering higher education with comparable qualifications (Zwysen and Longhi 2018; Pilkington 2020; Taylor et al. 2021; Seuwou et al. 2023). These patterns reflect not individual deficits but structural exclusions, including experiences of microaggressions, lack of curricular representation, and weaker institutional support (Arday et al. 2022; Bhopal 2024b).

Efforts to address these disparities have gained traction, with some universities adopting race‐conscious strategies to reform teaching, assessment, and student support. However, these reforms remain partial and politically vulnerable. The White Paper's emphasis on integration, merit, and self‐responsibility—echoing the logick of the Sewell Report (Commission on Race and Ethnic Disparities (CRED) 2021)—implicitly denies the structural nature of racial inequality, recasting it as a matter of effort, culture, or individual aspiration. In doing so, it provides ideological cover for the erosion of equity programmes under the guise of fairness or ‘levelling up.’

This discursive shift aligns with what Bhopal and Pitkin (2020) describe as ‘performative’ equality work: symbolic initiatives, such as the Race Equality Charter launched in 2016 by Advance HE (Higher Education), which do not challenge underlying structures of power. If the political climate discourages open engagement with racism as systemic, universities may retreat from even modest commitments to racial justice. The risk, as noted by Bhopal (2024a), is that policy discourse becomes a tool not for change but for maintaining the status quo—recycling the language of inclusion while resisting redistributive action.

Moreover, the cultural politics of the White Paper—its framing of belonging as conditional, its problematisation of minoritised family formations, and its focus on economic productivity—echo wider narratives that pathologise racialised communities. This assimilationist vision dovetails with educational practices that penalise those who deviate from normative expectations of behaviour, expression, or knowledge (Gillborn 2008; Tikly 2022). For instance, the requirement for continuous employment and high English proficiency as conditions for settlement mirrors the kind of cultural filtering that many BAME students encounter in academic assessment and professional recruitment (Ingram and Allen 2019).

In this way, the White Paper functions not merely as immigration policy, but as a racialising apparatus—shaping who is visible, who is heard, and who is deemed worthy of full membership in the national and academic community. CRT reminds us that such policies cannot be understood as purely technocratic or administrative; they are embedded within broader racialised logics that structure state power and institutional practise. They are part of an evolving regime of racial capitalism, in which state institutions extract value from minoritised populations while simultaneously limiting their rights and recognition (Delgado and Stefancic 2023; Bhopal 2024b). From this perspective, the persistence of the awarding gap is not an aberration to be corrected by better data or new charters, but a symptom of how British higher education continues to reproduce social stratification through the very mechanisms it claims to transcend. It is important to note that the BAME students discussed here are not necessarily migrants. Rather, the argument connects these domestic patterns of racial inequality to the broader racialised logics of the immigration system, which similarly constructs value and belonging through metrics of performance and compliance.

#### 2.2.3 Knowledge Hierarchies, Staffing Inequalities, and Racialised Gatekeeping

The implications of the 2025 Immigration White Paper extend beyond student mobility and into the composition of academic labour itself. UK higher education institutions have long relied on internationally recruited staff—many of whom are early‐career researchers and lecturers from the Global South—whose intellectual contributions enrich institutional knowledge cultures (Minocha et al. 2018). For example, many early‐career international researchers employed in UK universities—including those from the Global South—earn salaries below the proposed £38,700 threshold, placing them at risk of ineligibility for skilled worker visas despite holding advanced qualifications (Sanderson 2024). While the threshold formally applies to roles within the UK, its impact is unevenly distributed, given that researchers from the Global South are more likely to occupy lower‐paid or temporary positions within British academia. The ostensibly objective use of ‘salary’ as a filtering criterion obscures how such policies function as racialised and classed proxies, selecting for certain forms of capital while disqualifying others.

In sociological terms, this filtering process reinforces global academic stratification, whereby Western qualifications and institutional affiliations are assigned higher epistemic value than those from the Global South (Demeter 2020; Ramadan 2025; Roberts 2013). The state's valorisation of ‘Global Talent’ is not merely a technical exercise in skill identification; it constructs a hierarchy of worth that aligns with colonial geographies of knowledge production. In doing so, it entrenches what Bourdieu would identify as symbolic violence—rewarding those who already possess legitimised forms of capital while devaluing other knowledges as inferior or irrelevant (Bourdieu 1986). The ethos of attracting the ‘brightest and best’ is thusly less an objective standard than a racialised discourse of desirability that privileges whiteness, elite anglophone credentials, and conformity to neoliberal models of productivity — that is, individualised, market‐driven measures of worth tied to employability, competitiveness, and economic utility (Tomlinson and Tholen 2023).

These exclusions are not abstract. The racial composition of the faculty affects both institutional culture and student experience. Numerous studies affirm that the presence of racially minoritised staff improves mentoring, role modelling, and student outcomes—particularly for students from similar backgrounds (Smith 2022). If restrictive immigration rules diminish staff diversity, this compounds the broader epistemic marginalisation already present in British academia. Moreover, the reduction of Global South voices in UK research spaces narrows the horizon of academic inquiry itself, limiting the scope of intellectual critique and cross‐cultural exchange at a time when global challenges—such as climate change, migration, and geopolitical conflict—demand more inclusive and interdisciplinary collaboration than ever before.

#### 2.2.4 Work Transitions, Racialised Capital, and the Stratification of Labour Markets

The racialised effects of immigration policy also manifest in the transition from education to employment—a site where formal inclusion often gives way to informal exclusion. A university degree is widely seen as a gateway to better labour market outcomes; yet for racially minoritised graduates, this promise remains unevenly fulfiled. A substantial body of evidence shows that even after controlling for education, minority graduates face higher rates of unemployment and underemployment compared to their white peers (Zwysen and Longhi 2018; Lessard‐Phillips et al. 2018). Access to elite internships, professional networks, and insider knowledge—what Bourdieu terms social and cultural capital—is unevenly distributed, disadvantaging students from racialised and working‐class backgrounds (Bourdieu 1986).

To mitigate these disadvantages, some universities have adopted targeted employability interventions. For example, the Be SMART programme—evaluated by Oputa and Cross (2021)—pairs BAME students with staff mentors to develop professional skills and self‐efficacy. Such initiatives attempt to compensate for the deficit in social capital that often separates racially minoritised students from their more privileged peers. However, these programmes exist within a broader policy climate that is increasingly hostile to race‐conscious interventions. The White Paper, like the Sewell Report before it, avoids any acknowledgement of racial inequality in either higher education or employment, instead foregrounding individual merit, integration, and national productivity as the guiding logics of immigration reform (Commission on Race and Ethnic Disparities (CRED) 2021; HM Government 2025).

This discursive silence is not incidental; it marks a withdrawal of state leadership on racial justice, thereby legitimising institutional inaction. Bhopal and Myers (2025) argue that minority ethnic students face compounded barriers not only during their time at university but also in their career aspirations—shaped by limited access to capital, institutional indifference, and the psychic burden of systemic exclusion. When government policy positions disadvantage as a failure of integration or resilience, it delegitimises structural critiques and reinforces deficit narratives.

The psychosocial dimensions of this exclusion are particularly salient for students from immigrant‐origin backgrounds. Research shows that children and young adults internalise the racialised hierarchies communicated by state discourse and immigration enforcement (Naseem 2019; Tikly 2022). When migrants are framed as burdens or rule‐breakers, and when families are subjected to punitive visa regimes, young people receive a powerful message about their conditional belonging. The Windrush scandal offers a paradigmatic example of this affective trauma—where the formal status of citizenship failed to protect Black Britons from bureaucratic violence (Gentleman 2019). Similarly, the exclusionary logics embedded in the 2025 White Paper risk reinforcing the sense amongst minoritised youth that they are permanent outsiders, subject to scrutiny, suspicion, and state‐sanctioned insecurity.

These affective conditions shape educational outcomes. Students whose identities and contributions are not recognised within the institutional culture may experience diminished agency and participation, limiting their ability to view education as empowering (Tikly 2022). Conversely, a civic culture that affirms their dignity and potential can foster belonging and aspiration. In this sense, immigration policy is not just about borders; it is about boundaries—of inclusion, legitimacy, and recognition. The 2025 White Paper, by invoking economic contribution and cultural conformity as the criteria for belonging, re‐inscribes a racialised regime of value that undermines both equality and education.

### 2.3 Racialised Labour Markets and the Stratifying Logick of Immigration Policy

#### 2.3.1 A Two‐Tier Workforce and the Political Economy of Migrant Stratification

The 2025 Immigration White Paper introduces a recalibrated work visa framework that, beneath its technocratic veneer, constructs and legitimises a two‐tier labour system underpinned by racialised hierarchies. By raising salary thresholds and narrowing eligibility for long‐term work migration to predominantly high‐skilled occupations—such as finance, engineering, academia, and IT—the policy elevates a professional class of migrants, disproportionately drawn from economically privileged, anglophone, or majority‐white nations (Kao and Thompson 2003; Heath and Di Stasio 2019). These individuals typically possess greater stocks of cultural capital and institutional legibility, enabling smoother pathways to visa acquisition, labour market insertion, and eventual settlement.

Conversely, the policy systematically curtails entry routes for migrants in manual, caregiving, and service‐based roles—sectors historically populated by racialised labour from the Global South and Eastern Europe (Virdee 2014; Wadsworth 2018). Recent scholarship demonstrates that East–West labour mobilities are themselves structured by racialised hierarchies: ‘Eastern Europeans’ are ambiguously racialised—proximate to ‘Europeanness’ yet positioned as inferior and disposable within Western labour markets—while bureaucratic bordering practices produce differential inclusion and long‐term precarity in care and service work (Lewicki 2023; Manolova 2022; Gawlewicz 2015). The introduction of the Temporary Shortage Occupation List, which offers tightly controlled, non‐renewable entry for sub‐graduate workers without settlement rights or family reunification entitlements, functions not as a mere labour‐market instrument but as a racialised filtration device. As the government explicitly states its intent to ‘restrict dependants for lower skilled workers’ (HM Government 2025), it simultaneously devalues the social reproduction and familial lives of such migrants—most of whom are women and men of colour working in care, agriculture, food processing, and cleaning (Migration Advisory Committee 2022; Booth 2024; Home Office 2024).

This bifurcation is not new; rather, it intensifies a long‐standing model of racial capitalism wherein migrant bodies from marginalised geographies are rendered disposable, extractable, and structurally excluded from national belonging (Virdee 2014; Samers and et al. 2016). By institutionalising temporary, conditional labour channels for essential yet undervalued roles, the policy reanimates a guest worker logick, constructing certain racialised groups as labouring subjects without civic rights or cultural legitimacy. In this schema, the production of value and the denial of settlement are co‐constitutive: the very sectors most reliant on racialised labour become sites of permanent precarity, legitimised through technocratic policy language. Such dynamics resonate with critical race accounts of institutionalised marginalisation, wherein technocratic discourses of efficiency and fairness sustain the reproduction of racially stratified outcomes (Solomos 2022).

#### 2.3.2 Economic Contribution, Meritocracy, and the Racialisation of Deservedness

The White Paper's emphasis on ‘earned settlement’ reiterates a familiar neoliberal doctrine of meritocracy, framing migration as an individualised exchange in which economic contribution begets civic recognition. Yet as Gillborn (2024) argues, the meritocratic promise remains illusory for many racialised individuals, who, despite qualifications and conformity, face entrenched disadvantage in UK labour markets. The policy's insistence on quantifiable metrics—salary thresholds, sustained employment, language proficiency—as proxies for belonging ignores the structural inequalities that mediate such outcomes.

Even when migrants meet formal integration criteria, they may still be obstructed by racial bias in hiring, undervaluation of foreign credentials, and limited access to elite networks (Zwysen and Longhi 2018; Miller et al. 2025). Foster and George (2025) document how skilled Black African and Caribbean migrants are frequently underemployed, while the UK‐born second generation continues to encounter ‘ethnic penalties’ despite full cultural and linguistic assimilation — a pattern confirmed by Zwysen and Longhi (2018), who found that second‐generation ethnic minority graduates in the UK face persistent employment and earnings gaps even after accounting for qualifications and background.

By assigning value exclusively to those who can ‘contribute’ in narrowly economic terms, the White Paper enacts what Bourdieu (1986) might describe as a misrecognition of capital. Migrants from non‐Western countries often lack the dominant cultural and social capital that British employers reward—not due to deficit but due to institutional norms—and thusly are systematically filtered out, not only from elite labour but from eligibility for settlement. In this sense, the policy is not merely about who earns enough; it is about whose labour and life worlds are deemed intelligible, legible, and worth including.

#### 2.3.3 Racialised Discrimination and the Normalisation of Unequal Labour Markets

Although the 2025 Immigration White Paper ostensibly targets non‐citizen populations, its broader effects reverberate across the racialised labour market in ways that impact both migrant and minority ethnic citizens. UK‐born workers from Black, Asian, and minority ethnic backgrounds may not be directly subjected to immigration control, yet the policies advanced by the White Paper shape the structural conditions in which they seek employment. The assumption that reducing ‘low‐skilled’ migration will generate more opportunities for local workers—including unemployed minority citizens—is largely unsubstantiated. As Wadsworth (2018) highlights, immigration has not been shown to depress employment rates amongst domestic populations in the long term. Yet, such narratives persist, often acting as rhetorical distractions from the systemic racism and institutional inertia that continue to impede the advancement of racialised workers.

Indeed, employment discrimination against minority ethnic groups remains entrenched. Heath and Di Stasio's (2019) meta‐analysis, spanning nearly 5 decades of British labour market data, demonstrates that discriminatory hiring practices have endured with little meaningful decline. Employers frequently favour white British applicants over equally qualified Black African, Afro‐Caribbean, and South Asian candidates. Rather than addressing these barriers, the White Paper's silence on racial bias tacitly sustains a system in which structural inequality is reframed as individual failure. By positioning immigration reform as a substitute for anti‐discrimination measures, the policy elides the labour market's racial hierarchies, thereby reinforcing the status quo. A critical race reading reveals the ideological work being performed here: the White Paper recodes structural exclusion as a matter of national labour market optimisation, effectively displacing responsibility for racial injustice onto individuals seen as insufficiently skilled, integrated, or employable (Virdee 2014; Bhopal 2024b).

Moreover, the implementation of immigration checks—especially ‘Right to Work’ requirements—has extended the reach of border enforcement into the everyday experiences of racialised citizens. Although these policies are framed through formal compliance language, they have produced systematically discriminatory outcomes in practise. According to a study by the Joint Council for the Welfare of Immigrants (JCWI 2017), employers frequently admitted to avoiding applicants with foreign‐sounding names or those lacking British passports, for fear of breaching compliance rules. As Yuval‐Davis et al. (2019) argue, this phenomenon reflects the rise of ‘everyday bordering,’ wherein racialised individuals, even when citizens, are subjected to intensified scrutiny based on perceived foreignness. A British‐born man of Pakistani heritage, for instance, may find himself repeatedly asked to prove his right to work—an indignity rarely imposed on white peers. The White Paper's emphasis on stricter enforcement promises to deepen these dynamics, inadvertently incentivising discriminatory avoidance strategies by employers and increasing the psychic and material burdens borne by BAME citizens. Rather than eliminating unfairness, such measures exacerbate it, producing a climate in which racialised belonging is continually questioned and conditionally granted.

#### 2.3.4 Intersectionality, Gendered Stratification, and the Racial Politics of Labour Control

The White Paper's impact is not uniform across racialised groups; it is differentiated through the intersecting vectors of gender, immigration status, and socio‐cultural expectations. Minority ethnic women—particularly those from South Asian, African, and Caribbean backgrounds—experience some of the most acute disadvantages in the UK labour market. As Li and Heath (2020) show, Pakistani and Bangladeshi women face persistently low employment rates and earnings, even when controlling for qualifications and English language fluency. These disparities are compounded when immigration policy restricts access to dependant visas, curtails family reunification, or imposes linguistic and economic thresholds that disproportionately burden women in migrant households.

For example, policies limiting or eliminating spousal visas for Master's students and low‐skilled workers risk reinforcing patriarchal migration patterns and gendered roles, in which men migrate independently for work while women are left behind or rendered economically dependent. Where women do migrate, they may be classified as adult dependants and subjected to English language requirements (HM Government 2025). Although framed as integration tools, such measures can delay or obstruct family unity—especially for women from non‐English‐speaking contexts. The lived consequence may be that a highly educated woman, otherwise capable of economic contribution, is excluded from settlement or delayed from joining her spouse due to a technical language benchmark—effectively reducing her to a passive appendage of the primary visa holder.

This exclusion reflects a broader failure to acknowledge that integration is relational: it is not merely a question of migrant adaptation but of institutional inclusion and support. Without sufficient language training opportunities, community infrastructure, or recognition of caregiving responsibilities, the policy imposes asymmetrical demands that further marginalise women. As Miera (2012) observes, such integrationist paradigms often morph into assimilationist expectations, wherein migrants are required to perform cultural compliance without reciprocal structural change by host institutions.

The cumulative effect is a gendered and racialised bifurcation of the labour market. Women from non‐anglophone and majority non‐white countries are subjected to a regime in which their worth is measured by an idealised image of economic productivity that rarely accounts for unpaid care work, interrupted career trajectories, or discriminatory labour markets. The White Paper's emphasis on ‘earning’ one's place is silent on the structural barriers that make earning unequally distributed. In so doing, it amplifies what Gillborn (2024) critiques as the meritocracy myth—a narrative that obscures how race, gender, and class stratify access to opportunity.

Finally, even integration initiatives referenced in the White Paper risk veering into cultural disciplining—that is, the imposition of dominant cultural norms and values as prerequisites for social acceptance. The invocation of ‘British values’ and ‘social cohesion,’ without a clear commitment to anti‐racism, raises concerns that diversity will be welcomed only on assimilationist terms. Previous policies such as the Prevent strategy—introduced in 2006 as part of the UK government's counter‐terrorism agenda—have already shown how such logics can result in Muslim citizens and migrants being surveilled, silenced, or pathologised in their workplaces and educational institutions (Reed and Rees 2024). Unless integration programmes are accompanied by robust anti‐discrimination enforcement and institutional reform, they risk operating as soft mechanisms of control—ensuring not that minorities belong, but that they conform.

### 2.4 Reimagining Migration Governance Through a Racial Capitalist Lens

The 2025 Immigration White Paper, framed as a technocratic exercise in economic optimisation, must be understood not as a purely administrative intervention but as a policy artefact embedded within—and productive of—racialised hierarchies.

By treating migration as a matter of skills, salary, and cultural assimilation, the policy reifies longstanding structures of exclusion, repackaged in the language of merit and contribution. What appears as administrative reform is, in effect, a recalibration of the state's racialised bordering regime—one that codifies whiteness, Western credentials, and neoliberal subjectivities as the normative standard for migrant inclusion.

This paper has argued that such policies cannot be disaggregated from the broader social landscape in which they operate—a landscape already stratified by entrenched educational and labour market inequalities. In higher education, the emphasis on individual performance and integration occludes the institutional responsibilities that perpetuate differential attainment and belonging. As shown by Pilkington (2020) and Seuwou et al. (2023), addressing educational disparities requires structural transformation—not merely the compliance of the racialised subject. Yet the White Paper's silence on institutional racism, even as it gestures towards integration, suggests a policy imaginary that sees inequality as a problem of migrant deficits rather than systemic exclusion.

Similarly, in the labour market, the foregrounding of economic contribution erases the conditions under which racialised workers are systematically devalued. As Heath and Di Stasio (2019) and Gillborn (2024) demonstrate, racial bias continues to shape recruitment and progression, rendering the very notion of meritocracy suspect. By failing to address these embedded inequities, the White Paper transforms unequal outcomes into grounds for exclusion, enshrining disadvantage as disqualification. The migrant who is underpaid not because of lack of skill, but because of racialised devaluation, becomes ineligible for settlement—an institutional alchemy through which racial capitalism naturalises disposability.

In this context, the policy's façade of technocratic fairness collapses. Rather than levelling the playing field, it recalibrates it in favour of those already proximate to whiteness, cultural capital, and institutional legibility. Migrants from the Global South, particularly women and those entering low‐paid sectors, are cast as surplus labour—welcomed temporarily, yet structurally denied long‐term inclusion. As Bourdieu (1986) reminds us, capital is not merely material but symbolic; and the denial of settlement rights on the basis of undervalued work, foreign qualifications, or limited English proficiency is not simply an economic judgement but a sociocultural one—marking certain bodies as unworthy of permanence.

Moreover, by reinforcing assimilationist demands while neglecting the racism embedded in host institutions, the White Paper perpetuates a one‐way model of integration. Language acquisition, economic productivity, and social conformity are imposed as individual obligations, without corresponding institutional commitments to equity or anti‐racism. This mirrors what Tikly (2022) critiques in educational contexts: that colour‐blind universalism not only ignores racial disparity but often entrenches it. A genuinely just migration regime would entail reciprocal transformation—demanding not only that migrants adapt, but that institutions become more just, inclusive, and reflexive.

Reform, then, must proceed from an anti‐racist paradigm. This includes conducting robust Racial Equality Impact Assessments, embedding anti‐discrimination enforcement into immigration governance, and recognising migrants as social and civic actors—not merely economic inputs. Policies must be evaluated not solely by their capacity to ‘control’ migration flows, but by the kind of society they reproduce. Immigration policy, as this paper has argued, is not a peripheral administrative concern—it is central to the ongoing (re)formation of national belonging, worth, and legitimacy.

To that end, aligning migration reform with racial justice agendas in higher education and employment is not aspirational but essential. The institutional responses outlined by Bhopal (2024a),  (2024b), Miller et al. (2025), and Taylor et al. (2021)—from decolonising curricula to enforcing pay gap transparency and affirmative hiring—offer a template for how integration might be reimagined as institutional accountability rather than migrant compliance. Crucially, this means rejecting the zero‐sum logick that pits immigration control against equity. A society genuinely committed to fairness must ensure that the governance of borders does not come at the expense of those already marginalised within them.

Ultimately, the Immigration White Paper must be understood not as a discrete policy episode, but as part of a longer trajectory of statecraft through which racial capitalism is sustained. As Gentleman (2024) and Li and Heath (2020) observe, immigration law has long functioned as a sorting mechanism—one that disciplines, excludes, and stratifies according to race, class, and national origin. This analysis identifies three interlocking mechanisms through which immigration governance reproduces hierarchy: the translation of economic value into moral worth, the framing of structural inequity as individual deficit, and the normalisation of whiteness as the implicit benchmark of belonging. Recognising these dynamics establishes immigration policy as a central technology of racial statecraft and calls for renewed engagement from both sociologists and policymakers. For sociology, this entails interrogating policy as a site where symbolic and material hierarchies are continually produced, and extending comparative, interdisciplinary research that connects migration, higher education, and labour regulation. For policymakers and institutions, it requires moving beyond technocratic framings of immigration to confront how governance practices sustain racialised inequality. For anti‐racist praxis, it calls for transformative policy frameworks grounded in equity, solidarity, and repair—approaches that demand structural change rather than compliance from those already marginalised. Framed in this way, the paper advances a forward‐looking agenda for both sociological inquiry and anti‐racist policymaking.

## Funding

The authors have nothing to report.

## Conflicts of Interest

The authors declare no conflicts of interest.

## Data Availability

The authors have nothing to report.
